# The Content, Quality, and Behavior Change Techniques in Nutrition-Themed Mobile Apps for Children in Canada: App Review and Evaluation Study

**DOI:** 10.2196/31537

**Published:** 2022-02-16

**Authors:** Jacqueline Marie Brown, Beatriz Franco-Arellano, Hannah Froome, Amina Siddiqi, Amina Mahmood, JoAnne Arcand

**Affiliations:** 1 Faculty of Health Sciences Ontario Tech University Oshawa, ON Canada

**Keywords:** mHealth, children, app quality, behavior change techniques, child nutrition, mobile apps, Canada, mobile phone

## Abstract

**Background:**

Children increasingly use mobile apps. Strategies to increase child engagement with apps include the use of gamification and images that incite fun and interaction, such as food. However, the foods and beverages that children are exposed to while using apps are unknown and may vary by app type.

**Objective:**

The aim of this study is to identify the app content (ie, types of foods and beverages) included in nutrition-themed apps intended for children, to assess the use of game-like features, and to examine app characteristics such as overall quality and behavior change techniques (BCTs).

**Methods:**

This analysis used a cross-sectional database of nutrition-themed apps intended for children (≤12 years), collected between May 2018 and June 2019 from the Apple App Store and Google Play Store (n=259). Apps were classified into four types: *food games* or nongames that included *didactic nutrition guides*, *habit trackers*, and *other*. Food and beverages were identified in apps and classified into 16 food categories, as recommended (8/16, 50%) and as not recommended (8/16, 50%) by dietary guidelines, and quantified by app type. Binomial logistic regression assessed whether game apps were associated with foods and beverages not recommended by guidelines. App quality, overall and by subscales, was determined using the Mobile App Rating Scale. The BCT Taxonomy was used to classify the different behavioral techniques that were identified in a subsample of apps (124/259, 47.9%).

**Results:**

A total of 259 apps displayed a median of 6 (IQR 3) foods and beverages. Moreover, 62.5% (162/259) of apps were classified as food games, 27.4% (71/259) as didactic nutrition guides, 6.6% (17/259) as habit trackers, and 3.5% (9/259) as other. Most apps (198/259, 76.4%) displayed at least one food or beverage that was not recommended by the dietary guidelines. Food game apps were almost 3 times more likely to display food and beverages not recommended by the guidelines compared with nongame apps (β=2.8; *P*<.001). The overall app quality was moderate, with a median Mobile App Rating Scale score of 3.6 (IQR 0.7). Functionality was the subscale with the highest score (median 4, IQR 0.3). Nutrition guides were more likely to be educational and contain informative content on healthy eating (score 3.7), compared with the other app types, although they also scored significantly lower in engagement (score 2.3). Most apps (105/124, 84.7%) displayed at least one BCT, with the most common BCT being *information about health consequences*.

**Conclusions:**

Findings suggest nutrition-themed apps intended for children displayed food and beverage content not recommended by dietary guidelines, with gaming apps more likely to display not recommended foods than their nongame counterparts. Many apps have a moderate app quality, and the use of consequences (instead of rewards) was the most common BCT.

## Introduction

### Background

Establishing healthy eating patterns in early childhood promotes growth and development and reduces the risk of obesity and noncommunicable diseases [1-3]. This is also a time when habitual dietary patterns are established [4]. However, many children worldwide have poor quality diets [5-7]. In Canada, the average child has insufficient intakes of vegetables, fruit, and whole grain foods and consumes excess fat, sodium, and sugar [8,9], which is a dietary intake pattern strongly associated with childhood overweight, obesity, and chronic disease risk [10]. There are a multitude of factors that influence children’s dietary attitudes, behaviors, and food choices, including intrinsic (eg, predisposed biological tendencies and gender) and extrinsic factors to children, such as the family (eg, mealtime and parenting style) and the community (eg, schools and media) [11]. Several strategies have been developed to improve child healthy eating habits including family and school-based interventions [12], nutrition policies [13,14], and the promotion of nutrition education, food skills, and food literacy [15]. These interventions can have profound health system and economic advantages [1,16].

Technology has evolved into a central part of everyday life [17,18]. In Canada and before the COVID-19 pandemic, over 25% of children spent more than 2.5 hours each day in front of a screen [19], with 99% of them having internet access outside of school. Almost a quarter of the children in grades 4 and 5 and half of the children in grade 7 owned a smartphone [20]. Studies have also demonstrated that children as young as 3 and 4 years old use their parents’ smartphones between 25 and 50 minutes a day to watch television and videos, listen to music, and play games [20-23]. Children respond positively to fun and engaging challenges [24] that are captivating and motivating [25]. It is therefore understandable why games, advergames, and other digital activities are highly popular among children [26] and are substantively added to the digital marketplace [27].

Mobile health (mHealth), defined by the World Health Organization “as medical and public health practice supported by mobile devices, such as mobile phones, patient monitoring devices, personal digital assistants (PDAs), and other wireless devices” [28], is a convenient approach to support health promotion [29] and nutrition education among adults and children [17]. In particular, mHealth interventions have the potential to better engage youth about health-related topics, compared with traditional health interventions [17]. Among youth, mHealth apps have been found to increase fruit and vegetable intake [30-33], improve nutrition knowledge [32-34], promote food choice awareness and healthy eating behaviors [35,36], reduce sugar intake [37], and improve physical activity [38]. Furthermore, mHealth apps, as part of multicomponent interventions, can be effective tools to improve and support health-related behaviors [38]. Previous research has demonstrated that app quality, such as the ability to customize an app and ease of use, influence the overall effectiveness of mHealth apps on health and behavioral outcomes [39-41]. Additional evidence indicates that the integration of appropriate behavior change techniques (BCTs) [42] into mHealth apps further enhances their effectiveness [39,42,43]. Although there is strong evidence to support the efficacy of mHealth apps designed and evaluated by health researchers, minimal research exists on the content, quality, and use of evidence-based BCTs of publicly available mobile apps with health-related content.

Given its relevance, there has been increasing interest in studying the use and content of mobile apps and mHealth interventions that are available to the public [22,39,44-46]. For example, one study showed that diet and nutrition apps have a higher proportion of advertisements in comparison with other general health and wellness apps [22]. Reviews of pediatric weight management, healthy eating, and physical activity mobile apps found that most lacked any integration of expert recommendations [44], and less than 1% underwent scientific evaluation [47]. Another study found that children and adolescents are frequently exposed to the advertisement of unhealthy foods when using social media apps [45]. However, the specific foods and beverages displayed in mobile apps intended for children that are not from advertisements have not yet been examined [38]. Even less is known about foods and beverages displayed in nutrition-themed apps that contain the highly engaging game-like features that attract children and youth. This concept is highly relevant as not all nutrition-themed apps are considered mHealth apps. In addition, with new apps becoming available almost every day, it has become difficult for users, as well as for health professionals and researchers, to identify, evaluate, and use high-quality mobile apps to support healthy habits.

### Objectives

The aims of this study are 3-fold. First, we identified the app content (foods and beverages) included in different types of nutrition-themed apps intended for children and determined whether nutrition-themed apps with gaming features displayed more foods and beverages not recommended by dietary guidelines compared with nutrition-themed nongame apps. Second, we evaluated the overall quality of these apps using the Mobile App Rating Scale (MARS) [48], which is a “validated multidimensional measure of quality indicators” [48,49]. We also determined if app quality differed across the different types of nutrition-themed apps. Finally, we identified the different BCTs used in these apps, guided by a well-established taxonomy of such techniques [50].

## Methods

### Study Design

This research was a cross-sectional study that used a systematic search strategy and standardized evaluation process, modeled after comparable studies [51,52]. The Strengthening the Reporting of Observational Studies in Epidemiology checklist is presented in Multimedia Appendix 1.

### App Selection

#### Eligibility Criteria

Apps were eligible for inclusion in the analysis if they contained nutrition content relevant to children (ie, not targeted to parents), were rated by the app developer as being appropriate for an audience aged ≤12 years, were in the English language, were accessible to any user (ie, did not require an access code to use), were not affiliated with a brand or product, and were updated in the past 2 years. Excluded apps were simple food tracking apps (eg, calorie counting), chronic disease management apps (eg, diabetes), and apps that contained nutrition and food content irrelevant to dietary behaviors or education (eg, restaurant-themed time management games and word searches).

#### App Search Strategy

Between May 2018 and June 2019, apps were identified from the Canadian Apple App Store and Google Play Store, which are app retailers containing the greatest number of publicly available apps in Canada [27]. The search methodology used in this study was adapted from comparable studies that used multiple keywords and terms to conduct their searches [51,52]. A search of app categories was conducted using 16 unique search terms as follows: nutrition game, eating game, diet game, food education, food game, nutrition education, child nutrition, kids nutrition, kids food, kids healthy eating, health food, child health, kids health, health game, health education, and child education. The data extracted from the identified apps were title, developer, number of downloads (when available), and cost. All app information was entered into a database that was used throughout the screening, selection, and evaluation processes.

#### App Screening, Selection, and Classification

Identified apps first underwent screening for inclusion by 2 independent reviewers based on the title and developer. Duplicate apps, defined as apps appearing in both the Apple App Store and the Google Play Store, were identified and removed. Apps identified as relevant after the screening phase underwent a second independent review based on the detailed app description available in the respective app store. Those detailed descriptions determined if apps were eligible for inclusion in the analysis. Apps with a cost were purchased if they met the inclusion and exclusion criteria after reviewing the detailed descriptions. Apps were excluded from the study if they were removed from the marketplace before evaluation or if technical errors prevented a full evaluation (eg, app crashing). Disagreements at any stage of the app screening and evaluation process were resolved by a third independent reviewer.

The 4 app classifications (app types) were created using an inductive approach, which considered common app themes and characteristics observed during the review of apps, and 4 distinct classifications of apps were defined (Table 1). On the basis of their primary purpose, format, and core features, selected apps were classified into those four app types by two independent reviewers: food game, didactic nutrition guide, habit tracker, or other. Food games were defined as those that have implemented gamification techniques, such as rewards and competition, to engage the user in play involving food icons [53]. Nongame apps included didactic nutrition guides that provided information on food and nutrition to the user in written and picture format; habit trackers enabled users to log their food or drink intake [54] and apps classified as *other* did not contain any of the features nor had the primary purpose of the aforementioned app types.

**Table 1:** App types, definitions, and examples.

App type		Definition	Examples
**Food game**		An app that implemented gamification techniques, such as rewards and competition, to engage the user in play involving food icons	Dr Panda Restaurant 2 by Dr Panda Ltd; Strawberry Shortcake Bake Shop by Budge Studios
**Nongame**			
	Didactic nutrition guide	An app that provided information on food and nutrition to the user in written and picture format	Nutrition Lookup by SparkPeople; SuperFoodsRx—Essential Guide by SuperFoods Partners, LLC
	Habit tracker	An app that enabled users to log their food or drink intake	Fooducate—Nutrition Tracker by Fooducate, Ltd; Water Drink Reminder by Leap Fitness
	Other	An app that did not contain the features or served the purpose of a food game, didactic nutrition guide, or habit tracker	Food & Cooking Genius by Brainscape; LaLa Lunchbox by LaLa Lunchbox

#### App Evaluation

Each app was downloaded and used for approximately 5 to 10 minutes for the reviewer to fully evaluate all aspects of the app content. In-app purchases were not evaluated in apps as these additional costs were determined to be largely inaccessible to the target audience of children. Apps were reviewed by 2 independent reviewers, and disagreements were resolved in consultation with a third independent reviewer.

### App Content Assessment

#### Foods and Beverages Displayed

Foods and beverages displayed in apps were identified and classified into 16 food and beverage categories based on Canadian dietary guidelines, specifically the Canada’s Food Guide (CFG) [55] and Canada’s Dietary Guidelines [56]. Using the CFG, food and beverage categories were further classified as recommended (ie, foods that should be consumed more often) and not recommended (ie, foods that should be limited):

- Recommended food and beverages: fruit, vegetables, whole grain foods, unprocessed meat, fish, meat alternatives, milk products, and milk alternatives
- Not recommended food and beverages: refined grain foods, sugar drinks, desserts, chocolate and candies, salty snacks, pizza, fast foods, and processed meat

Apps were also assessed to determine if foods and beverages differed between *food game* apps and those without gaming features (ie, didactic nutrition guide, habit tracker, and others). In addition, textual healthy eating messages, such as “eat as many different colors as you can at each meal,” “eat breakfast every day, breakfast gives you energy and helps you think and learn” and “make at least half of your grain products whole grain each day,” were also identified in the apps.

#### Other Content Information

The number of app downloads and app cost were also extracted from the Apple App Store or Google Play Store. The number of downloads was only extracted from apps available in the Google Play Store, as information on the number of downloads was not available from the Apple App Store.

### App Quality Assessment

App quality was determined for the apps using the MARS [48]. The MARS contains 23 items divided into 5 subscales that also contain specific domains. Subscales of engagement (entertainment, interest, customization, interactivity, and target group), functionality (performance, ease of use, navigation, and gestural design), aesthetics (layout, graphics, and visual appeal), and information (accuracy of app description, goals, quality of information, quantity of information, visual information, credibility, and evidence base) were used to assess the objective quality of included apps [48]. The fifth subgroup, subjective quality (Would you recommend this app? How many times do you think you would use this app? Would you pay for this app? What is your overall rating of the app?), was not included because the apps were evaluated by researchers, not by the target audience of children; therefore, the subjective scores would not reflect the views of the intended audience. Each domain was rated by researchers on a 5-point Likert scale: 1=inadequate, 2=poor, 3=acceptable, 4=good, and 5=excellent. If a domain was not present in the app, that domain was rated as *N/A* and was not included in the domain subscale score. The average of all scores from each evaluated domain was considered as the overall app quality (MARS).

### BCT Assessment

The use of different BCTs in apps was evaluated in a subsample of nongame nutrition apps, most likely to contain mHealth features, using the BCT Taxonomy (v1), developed by Michie et al [50]. This taxonomy identifies 93 hierarchically clustered techniques grouped within 16 behavioral clusters. Each app was evaluated for the presence or absence of each BCT listed in the taxonomy.

### Statistical Analyses

Data were tested for normality, and descriptive statistics were used to calculate the number and proportion of foods and beverages and other information displayed in the apps, both overall and by app type. To assess differences in foods and beverages between food game apps and nongame apps, data from didactic nutrition guides, habit trackers, and other apps were combined. A binomial logistic regression assessed whether food game apps displayed more foods not recommended by dietary guidelines compared with nongame apps. The proportion of foods and beverages in food game apps and nongame apps was calculated by food category and chi-square-tested for differences between both groups.

The median and IQR were calculated for the MARS score, subscales, and domains. The Kruskal-Wallis test was used to evaluate differences in the MARS scores and subscales between the 4 different app types. The frequency and proportion of use of the different BCTs were calculated by cluster label and by specific behavioral component for the subsample of apps using descriptive statistics. Statistical significance was set at *P*<.05, except for the between-group comparisons of the MARS score (and subscales) and the 4 different app types, where α was set at *P*<.01, to account for multiple comparisons. Statistical analyses were conducted using RStudio software (RStudio) [57].

## Results

### App Screening, Selection, and Classification

**Figure 1:**
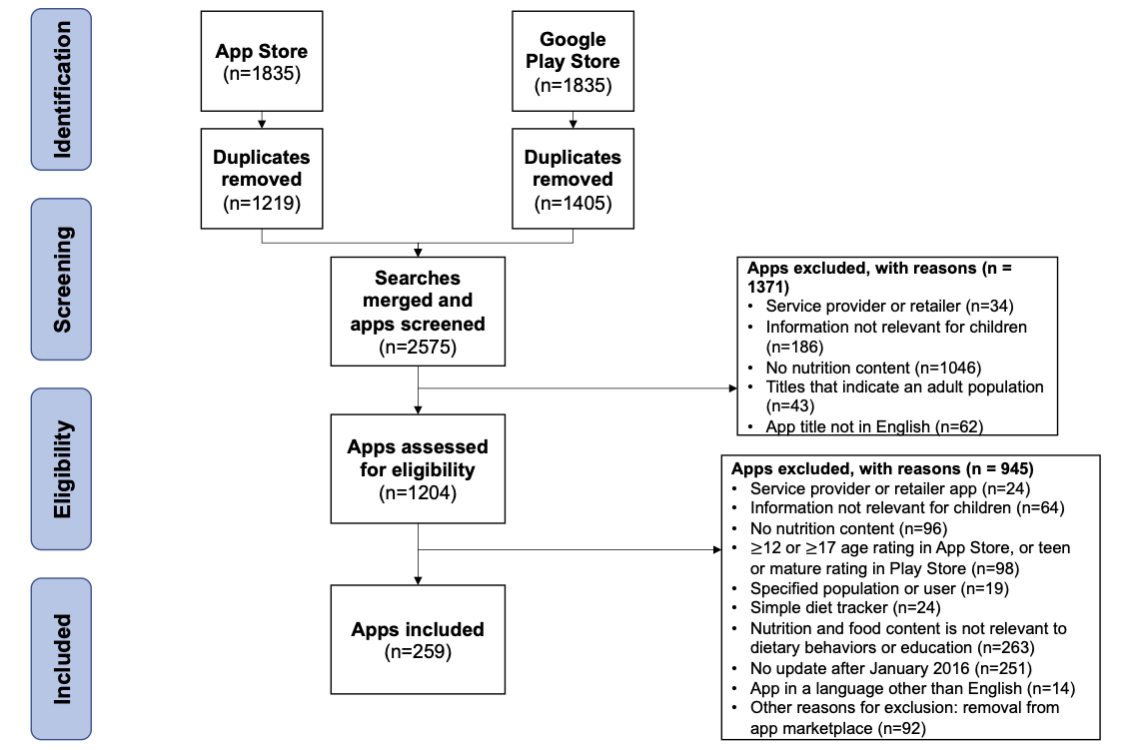
PRISMA (Preferred Reporting Items for Systematic Reviews and Meta-Analyses) flow diagram for used to identify nutrition-themed apps intended for children.

After removing duplicates, a total of 2575 unique apps were identified during the app search phase (Figure 1). From the 1204 apps that underwent title and developer screening review, 259 apps were eligible for inclusion in the analysis (interrater percent raw agreement=94.3% and Cohen κ=0.88). Multimedia Appendix 2 summarizes all included apps. Owing to the dynamic nature of the app marketplace, 60 apps were evaluated only by 1 reviewer, as these apps were removed from the marketplace during the app evaluation phase. For apps that were evaluated by 2 reviewers (199/259, 76.8%), app type classification yielded an interrater percent raw agreement of 91.7% and Cohen κ of 0.87. From the 259 apps, 162 (62.5%) were classified as food games, 71 (27.4%) as didactic nutrition guides, 17 (6.6%) as habit trackers, and 9 (3.5%) as other.

### App Content

Among all 259 apps included in this analysis, apps included a median of 6 (IQR 3) food and beverage items. The most prevalent food and beverage items in the apps were fruit (200/259, 77.2%), milk products (185/259, 71.4%), vegetables (179/259, 69.1%), and unprocessed meats (175/259, 67.6%). The least prevalent food items were salty snacks (61/259, 23.6%), fast foods (57/259, 22%), pizza (51/259, 19.7%), and milk alternatives (38/259, 14.7%; Table 2). Despite the high prevalence of foods recommended by dietary guidelines, only 28.9% (75/259) of the apps included explicitly healthy eating messages.

**Table 2:** Types of food and beverages displayed in nutrition-themed apps for children.

Food and beverages			Value, n (%)				
			All (n=259)	Food game (n=162)	Didactic nutrition guide (n=71)	Habit tracker (n=17)	Other (n=9)
**Presence of foods and beverages by category**							
	**Recommended by dietary guidelines^a^**						
		Fruit	200 (77.2)	110 (67.9)	68 (95.8)	14 (82.4)	8 (88.9)
		Vegetables	179 (69.1)	89 (54.9)	69 (97.2)	14 (82.4)	7 (77.7)
		Whole grain foods	81 (31.2)	13 (8)	53 (74.6)	14 (82.4)	1 (11.1)
		Unprocessed meats	175 (67.6)	95 (58.6)	62 (87.3)	13 (76.5)	5 (55.6)
		Fish	104 (40.2)	33 (20.4)	56 (78.9)	14 (82.4)	1 (11.1)
		Meat alternatives	110 (42.5)	29 (17.9)	63 (88.7)	14 (82.4)	4 (44.4)
		Milk products	185 (71.4)	110 (67.9)	56 (78.9)	14 (82.4)	5 (55.6)
		Milk alternatives	38 (14.7)	6 (3.7)	18 (25.4)	14 (82.4)	0 (0)
	**Not recommended by dietary guidelines^a^**						
		Refined grain foods	151 (58.3)	116 (71.6)	19 (26.78)	12 (70.6)	4 (44.4)
		Sugary drinks	92 (35.5)	62 (38.3)	15 (21.1)	15 (88.2)	1 (11.1)
		Desserts	103 (39.8)	75 (46.3)	10 (14.1)	14 (82.4)	4 (44.4)
		Chocolate and candies	133 (51.4)	108 (66.7)	9 (12.7)	14 (82.4)	2 (22.2)
		Salty snacks	61 (23.6)	38 (23.4)	6 (8.4)	14 (82.4)	3 (33.3)
		Pizza	51 (19.7)	29 (17.9)	7 (9.8)	14 (82.4)	1 (11.1)
		Fast foods	57 (22)	36 (22.2)	6 (8.4)	13 (76.5)	2 (22.2)
		Processed meats	81 (31.3)	57 (35.2)	8 (11.3)	13 (76.5)	3 (33.3)
**Displayed at least one food or beverage not recommended by dietary guidelines**							
	0 food and beverage		61 (23.6)	10 (3.9)	45 (17.4)	2 (0.8)	4 (1.5)
	≥1 food and beverage		198 (76.4)	152 (58.7)	26 (10)	15 (5.8)	5 (1.9)
Healthy messages			75 (28.9)	13 (8)	51 (71.8)	8 (47.1)	3 (33.3)
App with cost			35 (13.5)	12 (7.4)	16 (22.5)	3 (17.6)	4 (44.4)
Number of downloads			121 (46.7)	73 (45.1)	34 (47.9)	11 (64.7)	3 (33.3)

^a^Determined using categories and key messages provided by dietary guidelines (ie, Canada’s Food Guide and Canada’s Dietary Guidelines).

Overall, 46.7% (121/259) of apps had data available on the number of downloads. The median number of downloads was 500,000 (IQR 4,990,000), with a range of 50 to 89,000,000 downloads. Cost was evaluated for all apps, with 86.5% (224/259) of apps being free. The median cost for apps with a monetary charge was CAD $2.80 (US $2.24; 35/259, 13.5%), with a range between CAD $1 (US $0.80) and CAD $8.50 (US $6.80).

**Figure 2:**
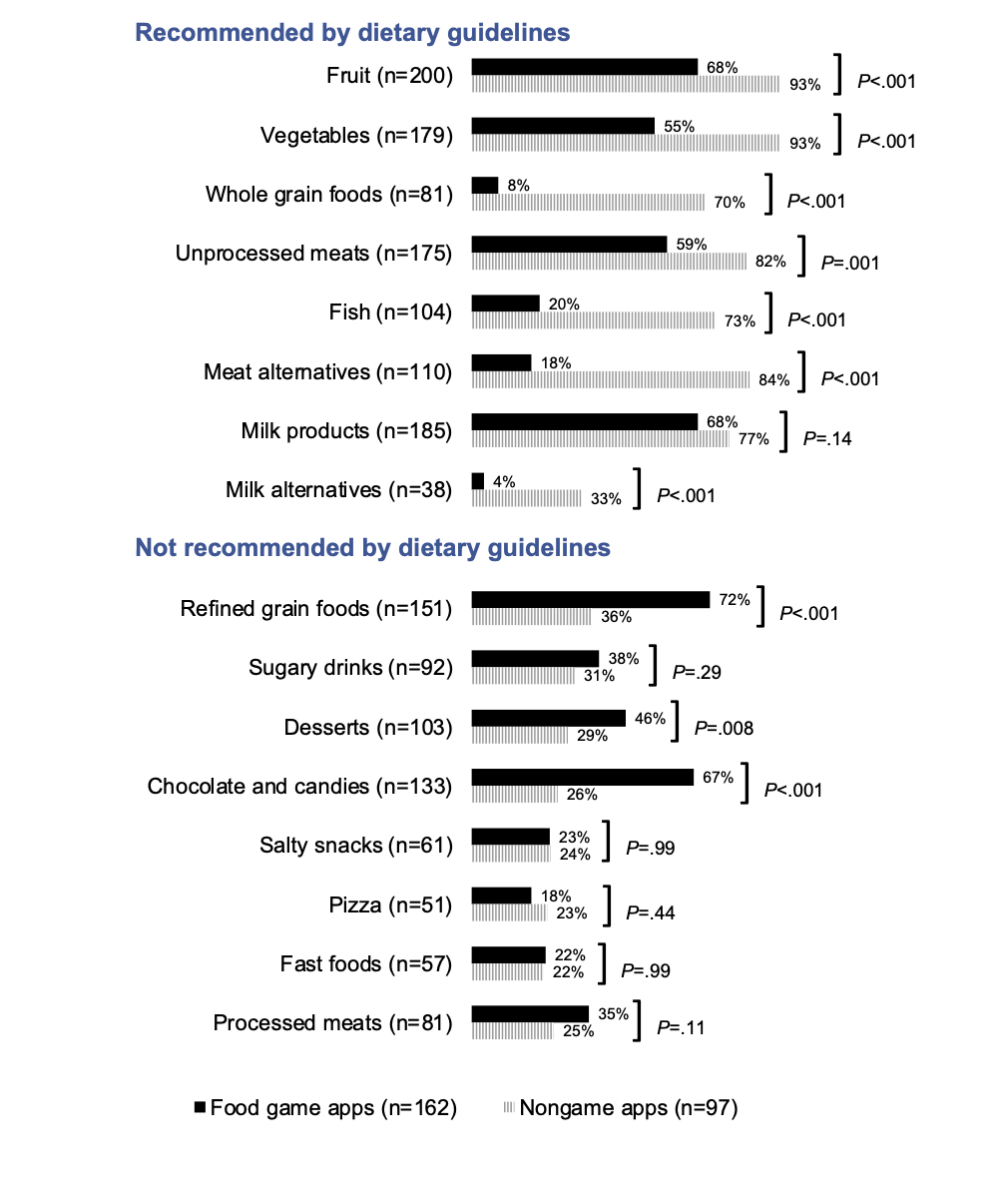
Proportion of foods and beverages displayed in food game apps and nongame apps by food category.

Food game apps, which comprised 62.5% (162/259) of apps overall, were almost 3 times as likely to display foods not recommended by dietary guidelines (β=2.8; *P*<.001), compared with nongame apps (ie, didactic nutrition guides, habit trackers, and other). In particular, high-sugar foods, such as chocolates and candies (*P*<.001) and desserts (*P*=.008) were significantly more likely to be displayed in food game apps, as shown in Figure 2 (detailed information in Multimedia Appendix 3). Importantly, food game apps also displayed significantly lower proportions of recommended foods and beverages in almost all recommended food categories, except for milk products, which was not significantly different between groups.

### App Quality

The overall app quality was moderate, with a median MARS score of 3.6 (IQR 0.7; Table 3). There were significant differences in overall quality between the 4 types of apps, with nutrition guides only having a median MARS rating of 3.2 (*P*<.001). Although nutrition guides were more likely to be educational and contain informative content on healthy eating (median score 3.7), compared with the other app types, they also scored significantly lower in overall engagement (score of 2.3) and in graphics and visual appeal (each with a score of 3). In addition, habit tracker apps were more likely to be engaging and aesthetic than were didactic nutrition guide apps. Overall, each subscale of the MARS was also moderate, with the highest ranked subscale being functionality (median 4, IQR 0.3), followed by aesthetics (median 3.7, IQR 0.7), information (median 3.6, IQR 1), and engagement (median 2.9, IQR 0.8). Although the information subscale received a moderate median MARS score, this average is likely magnified as many apps did not contain goals (234/259, 90.3%) or visual information (238/259, 91.9%), nor were they evaluated in available peer-reviewed literature (258/259, 99.6%). Overall, larger differences between app types were seen in the engagement and aesthetics subscales.

**Table 3:** Quality of nutrition-themed apps for children, overall, and by app type^a^.

App type		Value, median (IQR)						*P* value^b^
		All (n=259)	Food game (n=162)	Didactic nutrition guide (n=71)	Habit tracker (n=17)	Other (n=9)		
Mobile App Rating Scale		3.6 (0.7)	3.4 (0.5)	3.2 (0.4)	3.5 (0.3)	3.6 (0.7)	<.001	
**Engagement**		2.9 (0.8)	3.0 (0.8)	2.3 (0.7)	3.3 (0.5)	3.1 (1.4)	<.001	
	Entertainment	3 (1.5)	2.5 (2)	3.0(0)	3 (0.6)	3.5(1)	.02	
	Interest	3 (1)	3 (1.5)	3 (0)	3 (0.6)	3 (1)	.24	
	Customization	3 (1)	3 (0.5)	1 (2)	4 (0.5)	3 (3)	<.001	
	Interactivity	3 (1.5)	3 (0.5)	1 (2)	4 (0.5)	3 (2)	<.001	
	Target group	3.5 (1)	4 (0.5)	3 (1)	3 (0)	4.5 (1)	<.001	
**Functionality**		4.0 (0.3)	4.0 (0.4)	4.0 (0.3)	4.0 (0.3)	4.1 (0.8)	.01	
	Performance	4 (0.5)	4 (0.5)	4 (0)	4 (0.6)	4 (0.5)	.01	
	Ease of use	4 (0)	4 (0.1)	4 (0.5)	4 (0)	4.5 (1)	.002	
	Navigation	4 (0)	4 (0)	4 (0)	4 (0)	4 (1)	.03	
	Gestural design	4 (0.5)	4 (0.5)	4 (0)	4 (0)	4 (1)	.008	
**Aesthetics**		3.7 (0.7)	3.7 (0.7)	3.3 (0.5)	4 (0.6)	4 (0.3)	<.001	
	Layout	4 (0.5)	4 (0.5)	4 (0.5)	4 (0.5)	4 (0)	.007	
	Graphics	3.5 (1)	3.5 (1)	3 (0.5)	4 (0.6)	4 (0)	<.001	
	Visual appeal	3.5 (1)	3.5 (1)	3 (0.8)	4 (0.6)	4 (1)	<.001	
**Information**		3.6 (1)	3.5 (1.3)	3.7 (0.3)	3.5 (0.4)	3.3 (0.9)	.22	
	Accuracy	4 (0.5)	4 (0.5)	4 (0)	4 (0)	4 (0)	.19	
	Goals	3 (0.5)	3 (1)	3 (0)	3 (0.5)	3 (0.5)	.46	
	Quality	4 (0.8)	3 (0.1)	4 (0)	4 (0)	4. (0.5)	<.001	
	Quantity	4 (1)	3 (1)	4 (0.5)	3.5 (0.5)	3 (1)	<.001	
	Visual	4 (1)	3.5 (1)	3.5 (1)	4 (0)	4 (0)	.33	
	Credibility	1 (2)	1 (0)	3 (1)	3 (2)	1 (1.5)	<.001	
	Evidence-based^c^	N/A^d^	N/A	N/A	N/A	N/A	N/A	

^a^The 23-item Mobile App Rating Scale was used to assess the quality of the included apps on 4 subscales of engagement (5 domains), functionality (4 domains), aesthetics (3 domains), and information (7 domains). Each domain was rated on a 5-point Likert scale: 1=inadequate, 2=poor, 3=acceptable, 4=good, and 5=excellent. If a domain was not present in the app, that domain was rated as *N/A*. The average of all scores from each evaluated domain was considered as the overall app quality.

^b^Significant difference was set at *P*<.01 to account for multiple comparisons and determined by the Kruskal-Wallis test.

^c^Not evaluated as the number of responses was <5.

^d^N/A: not applicable.

### BCT Assessment

BCTs were identified in 84.7% (105/124 subsample) of apps that were evaluated. Among those, 72.4% (76/105) had 1 or 2 BCTs, 22.9% (24/105) had between 3 and 10 BCTs, and we also found 4.8% (5/105) of apps with more than 10 BCTs. The most common BCT clusters among the 105 apps were *natural consequences* (100/105, 95.2%), *shaping knowledge* (49/105, 46.7%)*,* and *goal setting and planning* (47/105, 44.8%). The most common individual BCTs identified in apps were *information about health consequences* (92/105, 87.6%), followed by *instructions on how to perform a behavior* (49/105, 46.7%; see Table 4 for detailed information)**.**

**Table 4:** Frequency of behavior change techniques (BCTs) identified in a sample of apps intended for children (n=105)^a^.

BCT cluster label and component			Frequency, n (%)
**Goal setting and planning**			47 (44.8)
	Goal setting (behavior)	16 (15.2)	
	Problem Solving	1 (0.9)	
	Goal setting (outcome)	12 (11.4)	
	Action planning	7 (6.7)	
	Review behavior goals	6 (5.7)	
	Review outcome goals	5 (4.8)	
**Feedback and monitoring**			36 (34.3)
	Feedback on behavior	11 (10.5)	
	Self-monitoring of behavior	12 (11.4)	
	Self-monitoring of outcome of behavior	10 (9.5)	
	Feedback on outcome of behavior	3 (2.9)	
**Social support**			3 (2.9)
	Social support (unspecified)	3 (2.9)	
**Shaping knowledge**			49 (46.7)
	Instruction on how to perform a behavior	49 (46.7)	
**Natural consequences**			100 (95.2)
	Information about health consequences	92 (87.6)	
	Information about social and environmental-consequences	2 (1.9)	
	Information about emotional consequences	6 (5.7)	
**Comparison of behavior**			11 (10.5)
	Demonstration of the behavior	11 (10.5)	
**Associations**			11 (10.5)
	Prompts cues	11 (10.5)	
**Repetition and substitution**			4 (3.8)
	Behavior substitution	2 (1.9)	
	Habit formation	1 (0.9)	
	Graded tasks	1 (0.9)	
**Comparison of outcomes**			4 (3.8)
	Credible source	4 (3.8)	
**Regulation**			2 (1.9)
	Reduce negative emotions	1 (0.9)	
	Conserving mental resources	1 (0.9)	
**Identity**			2 (1.9)
	Identification of self as role model	2 (1.9)	

^a^Identified using the behavior change technique taxonomy developed by Michie et al [50].

## Discussion

### Principal Findings

This study is among the first to identify and empirically evaluate foods and beverages displayed in nutrition-themed apps intended for children in publicly available app stores. Most apps displayed foods and beverages not recommended by dietary guidelines (especially among those apps with game-like features), which had a moderate app quality. Importantly, this study also identified the use of consequences, rather than rewards, as the most common BCT in apps most likely to contain mHealth features.

Apps displayed a median of 6 food or beverage items to children, and the majority (198/259, 76.4%) displayed foods and beverages not recommended by dietary guidelines. This finding is concerning as it is likely a conservative estimate of exposure to food products that are not recommended by dietary guidelines in apps. This study excluded apps from branded products and did not evaluate in-app advertisements by food companies, which could have largely increased the number of displayed foods and beverages that are not recommended by dietary guidelines [45]. It was not surprising that the types of foods and beverages differed by app type. For example, nutrition guides, which are informative and educational in nature, displayed more fruit, vegetables, meat alternatives, and milk products, whereas habit tracker apps displayed large amounts of both foods recommended and not recommended by dietary guidelines. In addition, the nutrition guide apps were found to be of lower overall quality, which was largely the result of having fewer engaging features and lower quality graphics and visual appeal. Of notable concern was that food game apps were more likely to display *unhealthy* foods to children, such as chocolates, candies, and desserts. They also displayed significantly fewer recommended foods compared with nongame apps. This finding concurs with other studies that have found that the food content in apps may not align with dietary recommendations and lack evidence-based health information [40,44,58,59]. Childhood is the formative life stage when food and nutrition preferences, attitudes, and habits are learned [13], which are traits known to carry over into adulthood [60]. The latter highlights the importance of enhancing healthy eating and nutrition knowledge at an early life stage [17]. As children are more likely to be exposed and influenced by web-based media platforms [26,61,62], especially high-quality and engaging apps [24], it is critical that app developers limit the use of *unhealthy* foods in mobile game apps for children [26,63,64]. Although nongame apps displayed food and beverage items that are recommended by dietary guidelines, their limited ability to engage users indicates that they are unlikely to positively influence healthy eating behaviors.

Although nutrition guides may benefit children from an educational standpoint, this study found that they may be less attractive and engaging for children based on their design features. This finding is relevant because there is significant potential for mobile apps that can be used to deliver engaging and high-fidelity interventions to educate and motivate children about healthy eating practices [65]. Several studies have emphasized the potential positive role of mHealth apps as cost-effective [46], low burden interventions to promote healthy eating, self-monitoring, and behavior change [66,67]. For instance, serious games (ie, digital games designed for educational purposes) have been found to support children’s increased vegetable and fruit intake [30-33], knowledge of macronutrients [32,33], food choice skills [35], and reduced sugar intake [37]. Moreover, healthy eating messaging can be incorporated into apps. Although our study found that only 8% (13/162) of food game apps contained messages aligned with dietary guidelines, 72% (51/71) of didactic nutrition guides and 42% (8/17) of habit trackers displayed healthy eating messages. These findings suggest that it is possible to use apps as a vehicle to support healthy eating messaging and nutrition education, as it has been used in other areas such as physical and mental health [68,69]. However, many apps on the marketplace would require modifications to align messaging with dietary guidelines and to include elements to increase their overall quality (visual appeal, graphics, engaging features, and BCTs). As nutrition guide and habit tracker apps may not be highly engaging for children, integrating gaming elements into these educational and informative apps may be more impactful in promoting the uptake of healthy eating knowledge and behaviors [41,70]. An example of a game-based nutrition education app is Foodbot Factory [71], which was designed to teach children about CFG and positively influenced children’s knowledge of CFG guidelines [34].

Importantly, the efficacy of mHealth interventions can be significantly impacted by overall app quality [48] and the integration of suitable theory-based BCTs [50]. The MARS, for instance, has been used to identify high-quality medication reminder apps [72]. However, a lack of initial and sustained engagement has also been identified as a key constraint that limits digital nutrition promotion interventions [73]. In this study, we found that the overall quality of the apps, as evaluated by the MARS, was moderate, with apps receiving a median quality score of 3.6/5. These results underline the need to integrate engagement and motivation cues, factors known to strongly influence how long children will interact with an app [24]. Most apps also used *information about health consequences* as a BCT, which seems to be an unsupported tactic to induce behavior change (and less useful among children), compared with incentivization of positive health behaviors (ie, the use of points and rewards) [74]. Furthermore, limited evidence exists on user testing [73] and the assessment of app effectiveness in terms of user satisfaction [22]. Thus, the development of effective and engaging mHealth apps not only requires evidence-based content and appropriate BCTs but also necessitates the feedback of end users and evaluation of effectiveness through appropriately designed studies.

### Limitations

There are limitations to this study. It may be argued that a search of this kind cannot be truly systematic because of the dynamic nature of the app marketplace and limited search and data extraction abilities [75,76]. However, the use of multiple search terms to identify apps allowed this search to reach saturation and capture the most common nutrition apps targeted to children. Although paid apps were included in this study when free apps contained in-app purchases, only the free content was analyzed because the use of in-app purchases is likely to be less accessible to our target audience of children and adolescents; this decision may have resulted in an underestimation of the foods and beverages included. In addition, for this analysis, information on data privacy was not assessed. Assessments of data privacy policies and procedures are critically important, especially when apps are targeted to children; however, this information was not publicly available at the time of data collection. Although the Apple App Store added information on data privacy in June 2020, the Google Play Store has not, as of January 2022. Finally, because of its cross-sectional design, this study does not allow us to determine the relationship between exposure to foods and beverages displayed in apps (particularly those not recommended by dietary guidelines) and children’s health, or their knowledge, attitudes, and behaviors related to such foods. However, describing the exposure is a first step to investigating this relevant topic, and this should be a focus of future research.

### Comparison With Prior Work

An earlier comparable study by Schumer et al [22] examined publicly available *diet* and *nutrition* apps available in the Google Play Store (n=86 apps that were relevant for any age group), identified the focus of apps (eg, education, tracking, and planning), diet types (eg, Paleo diet), and app features (eg, goal setting and feedback). In capturing this information, the authors described initial factors that potential users evaluate when deciding to use an app [22]. An Australian study used a comparable approach to evaluate food and nutrition-related mobile apps to support healthy family food provision [40]. This study found that apps targeting parents with children had an app quality score of 3.5, similar to our results; however, many apps also had poor engagement. This study builds upon the previous work by conducting an in-depth evaluation of the different foods and beverages displayed in apps and whether or not these foods and beverages met dietary guidelines. This study’s novelty further expands our understanding of apps intended solely for children and not parents or adults in general. In addition, our study goes beyond the findings of Schumer et al [22] in the assessment of apps intended for children by identifying those that were games, which is critical to examine as gaming apps are highly influential among youth [17,46]. A major strength of this study was the use of standardized evaluation and classification of apps, which was conducted independently by 2 reviewers, with a third independent reviewer to resolve disagreements. This process ensured a rigorous evaluation of the app content and classification. Another major strength was the use of the 2 major app stores, in contrast to the study by Schumer et al [22], which only involved 1 app store.

### Conclusions

This research demonstrated that nutrition-themed apps intended for children displayed many foods and beverages not recommended by dietary guidelines, and food game apps were more likely to display *unhealthy* foods and beverages compared with nongame apps. We also found that many of these apps in the subsample have a moderate app quality, and most of them use *information about health consequences* as a BCT. Nevertheless, given their popularity, nutrition-themed game apps have the potential to be used by health professionals, researchers, educators, and app developers to create evidence-based apps for children that align with dietary guidelines, which can be used to encourage healthy eating habits. Future research is required to broaden our understanding of how youth use and interact with apps containing nutrition content, their influence, and possible use for promoting nutrition education and healthy eating.

## Appendix

Multimedia Appendix 1 Strengthening the Reporting of Observational Studies in Epidemiology Statement—checklist of items that should be included in reports of observational studies.

Multimedia Appendix 2 Summary of nutrition apps included in the analysis (n=259).

Multimedia Appendix 3 Proportion of foods and beverages displayed in food game apps and nongame apps by food category (n=259).

## Abbreviations

BCT: behavior change technique
CFG: Canada’s Food Guide
MARS: Mobile App Rating Scale
mHealth: mobile health
