# Supplementary material for: The Content, Quality, and Behavior Change Techniques in Nutrition-Themed Mobile Apps for Children in Canada: App Review and Evaluation Study
Source: JMIR Mhealth Uhealth. 2022 Feb 16;10(2):e31537. doi: 10.2196/31537 (PMC8892278; doi:10.2196/31537)
Supplement: Multimedia Appendix 2 [file mhealth_v10i2e31537_app2.docx]

**Multimedia Appendix 2.** Summary table of nutrition apps included in analysis (n=259)

| **App Name** | **Developer** | **Cost** | **App Type** | **MARS Average** |
| --- | --- | --- | --- | --- |
| 10 Best Foods for You | Insplisity | None | Nutrition Guide | 2.71 |
| 5-2-1-0 Kids! powered by Henry Ford LiveWell | Pixofactor, LLC | None | Food Game | 3.64 |
| Amy's Donut - Baking Game | Games for Friends LLC | None | Food Game | 3.31 |
| Argus: Calorie Counter & Steps | Azumio Inc. | None | Habit Trackers | N/A |
| Baby cooking game: Free child early education game | yu zheng | None | Food Game | 3.96 |
| Baby Food Fair Chef | Kids Fun Club by TabTale | None | Food Game | 3.62 |
| Balance Diet | river studios | None | Nutrition Guide | N/A |
| BAM! Dining Decisions iPhone | Centers for Disease Control and Prevention | None | Food Game | 4.41 |
| Benefits of Eggs | JuBat | None | Nutrition Guide | 3.69 |
| Best Boiled Egg Diet Plan | Cylonblast Mobile Apps | None | Nutrition Guide | 3.24 |
| Best Diet Foods | DaDo | None | Nutrition Guide | N/A |
| Best Diet Foods Healthy Living | Michael Quach | None | Nutrition Guide | 3.40 |
| BRAIN BOOSTING FOODS | AXON | None | Nutrition Guide | 3.03 |
| BreakFast Food Maker - Kitchen Cooking Mania Game+A11:AS13* | Family Kids Games | None | Food Game | 3.54 |
| Breakfast Food Maker Kids Games (Girls & Boys)* | Best Fun Games | None | Food Game | 3.31 |
| Brownie Maker - Kids Food & Cooking Salon Games* | Ninjafish Studios | None | Food Game | 3.38 |
| Burger | Magma Mobile | None | Food Game | 4.23 |
| Burger Deluxe - Cooking Games* | Bubadu | None | Food Game | 3.50 |
| Burger Shop - Kids Cooking* | K3Games | None | Food Game | 3.42 |
| Cake Bites Maker | Bake More Cake Maker Inc. | None | Food Game | 3.65 |
| cake maker - cooking game* | Bubadu | None | Food Game | 3.32 |
| Cake Maker - Cooking games.* | Pazu Games Ltd | None | Food Game | 3.46 |
| Cake Maker Kids (Ads Free) | Bubadu | None | Food Game | 3.36 |
| Cake Pop Cooking! | Kids Food Games Inc. | None | Food Game | 3.07 |
| Cake Pop Maker* | Bake More Cake Maker Inc. | None | Food Game | 3.58 |
| Cake Pops Halloween Kids FREE | Beansprites LLC | None | Food Game | 3.07 |
| Calcium Counter and Tracker for Healthy Food Diets | First Line Medical Communications Ltd | Paid | Nutrition Guide | 3.13 |
| Calorie Counter - MyFitnessPal | MyFitnessPal, Inc. | None | Habit Trackers | 3.71 |
| Calorie Counter - MyNetDiary | MyNetDiary.com | None | Habit Trackers | 3.45 |
| Calorie quiz: Food and drink | Lemmings at work | None | Other | 3.13 |
| CalorieGuide Food Nutrition Facts Calculator for Fresh Produce & Healthy Diet Living | Jommi UG (haftungsbeschrankt) | Paid | Nutrition Guide | 3.22 |
| Candy Dessert Making Food Games for Kids | VZO entertainment | None | Food Game | 3.50 |
| Candy Maker Food Games-iMake Lollipops for Kids | Cubic Frog Apps | None | Food Game | 3.46 |
| cheese pizza lunch box - cooking game for kids | FROS studios | None | Food Game | 3.07 |
| Chef Kids - Cook Yummy Food* | TabTale | None | Food Game | 3.96 |
| Chewing Gum Maker - Kids Dessert Maker Games FREE | Beansprites LLC | None | Food Game | 3.21 |
| Chewing Gum Maker 2 - Kids Bubble Gum Maker Games | Beansprites LLC | None | Food Game | 3.35 |
| Chicken Gravy Maker - Cooking Game | 2d fun zone | None | Food Game | 2.77 |
| Chinese Food - Lunar New Year!* | Crazy Cats | None | Food Game | 3.27 |
| Chinese Food Maker! Food Games!* | himanshu shah | None | Food Game | 3.23 |
| Chinese Food! Make Yummy Chinese New Year Foods! | Kids Food Games Inc. | None | Food Game | 3.00 |
| Chocolate Bar Maker | Bluebear Technologies Ltd. | None | Food Game | 3.54 |
| Chocolate PiÃ±ata Cake Maker - Kids Dessert Food | Beansprites LLC | None | Food Game | 3.50 |
| Cook Baked Lasagna* | bweb media | None | Food Game | 3.77 |
| Cookie & Cake Maker Chef Game | Nikhil Khoda | None | Food Game | 3.23 |
| Cookie Candy Maker - Food Kids Games Free! | Best Fun Games | None | Food Game | 3.23 |
| Cookie Creator - Kids Food & Cooking Salon Games | Ninjafish Studios | None | Food Game | 3.23 |
| Cookie Dessert Maker - Food Kids Games! | Best Fun Games | None | Food Game | 3.38 |
| Cookie Doodle | Shoe the Goose | Paid | Food Game | 3.81 |
| Cooking Breakfast | Bubadu | None | Food Game | 3.85 |
| Cooking Food Maker Games for Kids (Girls & Boys)* | Quicksand Playground | None | Food Game | 3.38 |
| Cooking Food Making Kids Games (Girl Boy)* | Best Fun Games | None | Food Game | 3.35 |
| Cooking game - chef recipes | LPRA STUDIO | None | Food Game | 3.42 |
| Cooking Game - Hot Dog Deluxe | Bubadu | None | Food Game | 3.92 |
| Cooking Ice Cream Sandwiches | bweb media | None | Food Game | 3.54 |
| Cooking in the Kitchen | Girl Games - Vasco Games | None | Food Game | 4.23 |
| Cooking Your Fajitas | bweb media | None | Food Game | 3.35 |
| Cotton Candy Maker - Kids Cooking Games for Free | Muhammad Ishaque | None | Food Game | 3.38 |
| Cotton Candy Maker Kids FREE | Beansprites LLC | None | Food Game | 3.36 |
| Cotton Candy!* | Bluebear Technologies Ltd. | None | Food Game | 3.62 |
| Crazy Cookie Maker: Easy Baking For Kids | Adam Irvine | None | Food Game | 3.42 |
| Crazy Pizza Chef* | Kids Fun Club by TabTale | None | Food Game | 3.42 |
| Crazy Pizza Maker | Jignesh vataliya | None | Food Game | 3.46 |
| Cupcake Creator - Kids Food & Cooking Salon Games* | Ninjafish Studios | None | Food Game | 3.15 |
| Cupcake Food Maker Cooking Game for Kids* | VZO entertainment | None | Food Game | 3.27 |
| Daily Health Tips | Karthik App Solution | None | Nutrition Guide | N/A |
| Dessert Food Maker - Cooking Kids Games Free!* | Best Fun Games | None | Food Game | 3.08 |
| Dessert Slushy Maker Food Cooking Game - make candy drink for ice cream soda making salon! | Quicksand Playground | None | Food Game | 2.73 |
| Detox diet plan:Lose fat fast in 7 days | Patrikat Softech | None | Nutrition Guide | 3.47 |
| Diabetic Diet | Gato Apps | None | Nutrition Guide | 3.22 |
| Diabetic Diet Plan | Health & Fitness Corp | None | Nutrition Guide | N/A |
| Diet and Health - Lose Weight | tech.fit | None | Habit Trackers | N/A |
| Donut Maker! by Bluebear* | Bluebear Technologies Ltd. | None | Food Game | 3.42 |
| Dr. Panda Restaurant 2 | Dr. Panda Ltd | Paid | Food Game | 4.31 |
| Dr. Panda Veggie Garden | Dr. Panda Ltd | Paid | Other | 4.14 |
| Dr. Panda's Ice Cream Truck | Dr. Panda Ltd | Paid | Food Game | 4.23 |
| Drink Water Reminder N Tracker | Phoenix Games LLP | None | Habit Trackers | 3.33 |
| Dumb Ways JR Boffo's Breakfast* | Metro Trains Melbourne Pty Ltd | Paid | Food Game | 4.19 |
| Easy Vegetarian | HurryTheFoodUp | None | Nutrition Guide | 3.47 |
| Eat This Much - Meal Planner | Eat This Much Inc | None | Habit Trackers | 3.36 |
| Effective Weight Loss Guide | naveeninfotech | None | Nutrition Guide | 2.97 |
| Fair Food Donut Maker - Games for Kids Free | VZO entertainment | None | Food Game | 3.27 |
| Fair Food Maker Game* | Sunstorm Interactive | None | Food Game | 3.69 |
| Fat Burning Foods | Expert Free Apps Team | None | Nutrition Guide | N/A |
| Feed Them Right | C'TropFood | None | Food Game | 3.68 |
| Feed Twip - Kids learn how to cook healthy food | C'TropFood | None | Food Game | 3.91 |
| FitDash - Social Calorie, Activity and Nutrition Tracker | Cloforce LLC | Paid | Habit Trackers | 3.20 |
| Folate and B12 Counter & Tracker for Healthy Diets | First Line Medical Communications Ltd | Paid | Nutrition Guide | 3.13 |
| Food & Cooking Genius | Brainscape | None | Other | 3.65 |
| Food for Health (Health Benefits and Uses) | HR App Studio | None | Nutrition Guide | N/A |
| Food Game: better than diet | Cognifica/ Magic Gold, s.r.o. | None | Food Game | 3.33 |
| Food maker - dessert recipes* | LPRA STUDIO | None | Food Game | 3.38 |
| Food Making Kids Games & Maker Cooking* | Best Fun Games | None | Food Game | 3.31 |
| Food nutrition | kalash47 | None | Nutrition Guide | N/A |
| Food Nutrition Table | Seoptics | None | Nutrition Guide | 3.47 |
| Fooducate - Nutrition Tracker | Fooducate, Ltd. | None | Habit Trackers | 4.06 |
| FREE Cooking Food Maker Games For Kids & Baby Girl* | Fat Free Apps | None | Food Game | 3.31 |
| Fresh Food Runner | Alimentarium | None | Food Game | N/A |
| Fresh Salad Bar: Healthy Green Food making game for education & learning | Kashif Mumtaz | None | Food Game | 2.44 |
| Fruit Info Pro | Vasudev Me0n | None | Nutrition Guide | 2.60 |
| Fruits and Vegetables for Kids | ENYSOFT | None | Food Game | 2.62 |
| Fruits and Vegetables for Toddlers - Learning Game | romeLab | None | Food Game | N/A |
| Fruits and vegetables learning | Kids Learn With Fun | None | Food Game | 2.81 |
| FUNNY FOOD 2! Educational Games for Kids Toddlers!* | MAGE Studio Kid Games | None | Food Game | 4.04 |
| Garfield: My BIG FAT Diet | Crazy Labs | None | Food Game | 3.87 |
| Garfield's Defense 3: Diet Fight | Web Prancer/ Cosmos Epoch Investments Limited | None | Food Game | 3.67 |
| Gingerbread Crazy Chef - Cookie Maker* | Kids Fun Club by TabTale | None | Food Game | 3.58 |
| Glitter Food - Kids Café* | Junior Games Studio | None | Food Game | 3.50 |
| Gluco Bear | Eugene Bauer | None | Food Game | N/A |
| Gluten-Free Guide | Celiac Program - Children's National Health System | None | Nutrition Guide | 3.88 |
| Grandma's Kitchen | Fairlady Media | Paid | Food Game | 4.31 |
| Gummy Bear Maker Candy Design Game- iMake Gummies | Cubic Frog Apps | None | Food Game | 3.35 |
| Halloween Monster Food Maker - Vampire Party Night | Kids Food Games Inc. | None | Food Game | 2.79 |
| Happy Diet Endless Games | Ivan Lyudevig | None | Food Game | 3.29 |
| Happy Food - Healthy Food | Kokum Apps | None | Nutrition Guide | 3.90 |
| Happy Health - Healthy Eating Game! | Secret Attic Software Limited | None | Food Game | 3.38 |
| Happy Kids Meal Maker - Burger Cooking Game | Junior Games Studio | None | Food Game | 3.65 |
| Health and Nutrition | Gato Apps | None | Nutrition Guide | 3.20 |
| Health and Nutrition : Nutrition Food Guide | Adria Devs | None | Nutrition Guide | N/A |
| Health and Nutrition Guide | naveeninfotech | None | Nutrition Guide | 3.06 |
| Health Diet Foods Fitness Help | Data Recovery Software by RecoveryBull.com | None | Nutrition Guide | 3.07 |
| Health Nutrition - Fitness & Weight loss guide | Utility Expert | None | Nutrition Guide | N/A |
| Health Tips for Healthy Living | Michael Quach | None | Nutrition Guide | 3.50 |
| Health, Nutrition & Diet Guide | Organic Facts | None | Nutrition Guide | 3.53 |
| HealthifyMe: Lose Weight, Nutrition, Diet Plan | HealthifyMe | None | Habit Trackers | 3.76 |
| Healthy Breakfast Cooking Game* | ChaseZap | None | Food Game | 2.93 |
| Healthy Cooking Kitchen 17 | Chief Gamer | None | Food Game | 3.00 |
| Healthy Diet Help Guide FULL | SendGroupSMS.com Bulk SMS Software | None | Nutrition Guide | 3.09 |
| Healthy Eater by BabyBus | BABYBUS CO., LIMITED | None | Food Game | 3.18 |
| Healthy Eating Diet Food Game | BestopStudios | None | Food Game | N/A |
| Healthy Eating Kids Food Game | zineb chadali | None | Food Game | 3.83 |
| Healthy Eating Meal Planner & Clean Eating Recipes | Realized Mobile LLC | None | Nutrition Guide | N/A |
| Healthy Eating Meal Plans | Cooking recipes | None | Nutrition Guide | 3.53 |
| Healthy Food | OAJOO | None | Nutrition Guide | 4.13 |
| Healthy Food | Infinite | None | Nutrition Guide | 3.20 |
| Healthy Food for Fit | Ionic Webtech | None | Nutrition Guide | N/A |
| Healthy Food For Your Diet Plan | HLAPPS | None | Nutrition Guide | 3.20 |
| Healthy Guide and Nutrition | Alesha Production | None | Nutrition Guide | N/A |
| Healthy Nutrition Guide Pro | Cristina Gheorghisan | Paid | Nutrition Guide | N/A |
| Healthy Santa/Learn English/Healthy food | TECH SAVVY MINDS SRL | None | Food Game | 2.19 |
| Heart-Healthy Food Complete Guide | s_gambo | None | Nutrition Guide | 3.00 |
| Height Increase Diet Tips and Remedies Help | Kaveri Tyagi | None | Nutrition Guide | 2.94 |
| Hello Kitty Lunchbox | Budge Studios | None | Food Game | 4.65 |
| High Protein Diet | ChekarApps | None | Nutrition Guide | N/A |
| High School Lunchbox Food Chef | Family Kids Games | None | Food Game | 3.31 |
| Hotdog fever-Crazy Fast Food cooking fun & kitchen scramble game for Kids, Girls, Boys & Teens* | Kamran Abid | None | Food Game | 2.88 |
| Hungry Harry | Kunal Valrani | None | Food Game | 2.75 |
| Ice Cream Lollipop Maker - Cook & Make Food Games | Kids Food Games Inc. | None | Food Game | 3.36 |
| Ice Cream Maker Kitchen Games-iMake IcePops | Cubic Frog Apps | None | Food Game | 3.54 |
| Ice Cream Maker: Frozen Dessert Summer Cooking game | Kamran Abid | None | Food Game | 3.00 |
| Ice Cream Master: Free Food Making Cooking Games | Kids Food Games Inc. | None | Food Game | 3.43 |
| Ice Pop & Popsicle Maker | Ninjafish Studios | None | Food Game | 3.08 |
| Icy Dessert Maker - Frozen Ice Cream Treats* | Bake More Cake Maker Inc. | None | Food Game | 3.62 |
| Ingredients & Nutrition Dictionary | Edutainment Ventures- Making Games People PlayHealth & Fitness | None | Nutrition Guide | 3.19 |
| Ingredients-Nutritional Facts | Edutainment Ventures LLC | None | Nutrition Guide | N/A |
| Italian Food Chef Cooking | Zahid Sumra | None | Food Game | 3.77 |
| iTooch 6th Grade \| Health | eduPad Inc. | Paid | Other | 4.19 |
| Japanese Food Maker Food Games | himanshu shah | None | Food Game | 3.12 |
| Keto Diet for Beginners | Smad Studios | None | Nutrition Guide | 3.27 |
| Ketogenic Diet | Gato Apps | None | Nutrition Guide | 3.53 |
| Kids Cereal Maker - Candy & Dessert Cereal Games | Beansprites LLC | None | Food Game | 3.21 |
| Kids Diseases, Health and Growth tips | Zade Appz | None | Nutrition Guide | N/A |
| Kids Meal Maker - Lunch Food & Candy Cooking Game | Detention Apps | None | Food Game | 2.86 |
| Kitchen cooking Cash Register* | bobolink apps | None | Food Game | 3.12 |
| LaLa Breakfast: meal planning for kids | LaLa Lunchbox, LLC | Paid | Other | 4.33 |
| LaLa Lunchbox | LaLa Lunchbox, LLC | None | Other | 4.00 |
| Learn Diabetes, Cancer, and Nutrition by GoLearningBus | Quizmine.Com | None | Nutrition Guide | N/A |
| Lifesum - Diet Plan, Calorie Counter & Food Diary | Lifesum | None | Habit Trackers | 3.75 |
| Little Panda Chefâ€™s Robot Kitchen-Kids Cooking | BabyBus Kids Games | None | Food Game | 4.18 |
| Little Panda Gourmet* | BabyBus Kids Games | None | Food Game | 4.00 |
| Little Panda's Chinese Recipes | BabyBus Kids Games | None | Food Game | 4.29 |
| Magnesium Counter & Tracker for Healthy Food Diets | First Line Medical Communications Ltd | Paid | Nutrition Guide | 3.13 |
| Make Donut - Kids Cooking Game | K3Games | None | Food Game | 3.81 |
| Masha and the Bear: Food Games | Indigo Kids Limited | None | Food Game | 3.54 |
| Masterchef Cooking Games: Fun Restaurant & Kitchen | Touchzing Media | None | Food Game | 3.21 |
| Milkshake Maker - Kids Frozen Cooking Games | Ninjafish Studios | None | Food Game | 3.31 |
| Monash Uni Low FODMAP Diet | Monash University Low FODMAP diet team | None | Habit Trackers | 4.15 |
| More Blender | Maverick Software LLC | Paid | Food Game | 3.00 |
| More Snow Cones! | Maverick Software LLC | Paid | Food Game | 3.08 |
| My Baby Food - Cooking Game | Bubadu | None | Food Game | 4.38 |
| My Cake Shop HD - Cake Maker Game* | LAI SYSTEMS, LLC | None | Food Game | 3.46 |
| My Food - Nutrition for Kids | urbn; pockets | Paid | Food Game | 4.12 |
| My Food Guide | Health Canada \| Sant√© Canada | None | Nutrition Guide | N/A |
| My Panda Chef | BABYBUS | None | Food Game | 3.32 |
| My Pizza Shop - Pizza Maker Game* | TAPBLAZE | None | Food Game | 3.50 |
| My town kids School Lunchbox | FROS studios | None | Food Game | 3.38 |
| Noom: Health & Weight | 0om Inc. | None | Habit Trackers | N/A |
| Nutrition Lookup - SparkPeople | SparkPeople | None | Nutrition Guide | 2.91 |
| Nutritional Values | Tiny Di0saur Dev | None | Nutrition Guide | N/A |
| Onfit - Nutrition & Healthier & Lose Weight & Gain Muscle & Bariatric Surgery | Thiago Bernardes | Paid | Habit Trackers | N/A |
| Paleo Diet Recipes Made Easy | Trellisys.net | Paid | Nutrition Guide | 3.56 |
| Panda Chef, Chinese Recipes-Cooking Game for Kids | BabyBus Kids Games | None | Food Game | 3.71 |
| Papa's Freezeria To Go!* | Flipline Studios | Paid | Food Game | 4.08 |
| Pasta Crazy Chef - Make Your Own Mac and Cheese | TabTale LTD | None | Food Game | 3.22 |
| Peekaboo Fridge | Night & Day Studios, Inc. | Paid | Food Game | 3.11 |
| Pizza Maker Crazy Chef Game* | TabTale | None | Food Game | 3.62 |
| Potassium Counter & Tracker for Healthy Food Diets | First Line Medical Communications Ltd | Paid | Nutrition Guide | 3.13 |
| Potato Chips Factory for Kids-Kids Factory Game | KidsTech | None | Food Game | 3.15 |
| Protein Rich Food Source Guide | Kaveri Tyagi | None | Nutrition Guide | 3.09 |
| PUZZINGO Food Puzzles Game | 77Sparx Studio, Inc. | Paid | Other | 3.46 |
| R U Fueled - Nutrition tracker | Karina Kappel | Paid | Habit Trackers | N/A |
| Rainbow Ice Cream - Unicorn Party Food Maker | Kids Food Games Inc. | None | Food Game | 3.43 |
| Restaurant Kids Food Maker | romeLab | None | Food Game | 3.46 |
| Richard Pizza's Hungry Games | Intelligent Decision Systems, Inc. | None | Food Game | 3.57 |
| School Lunch Box - Lunch Box Maker | himanshu shah | None | Food Game | 3.42 |
| School Lunch Food * | YONGZHI QI | None | Food Game | 3.50 |
| School Lunch Food Maker 2: Free Cooking Games | Crazy Cats | None | Food Game | 3.43 |
| School Lunch Food Meal Maker - Candy, Burger, Toys* | Detention Apps | None | Food Game | 3.15 |
| School Lunch Maker - Kids Food & Snacks Games | Beansprites LLC | None | Food Game | 3.43 |
| School Meal Maker Lunch Food & Candy Cooking Game | Detention Apps | None | Food Game | 3.14 |
| Selenium Counter & Tracker for Healthy Food Diets | First Line Medical Communications Ltd | Paid | Nutrition Guide | 3.25 |
| Slushy Maker Spa | Ninjafish Studios | None | Food Game | 3.27 |
| Smoothie Master: A Crazy Chef Adventure | TabTale LTD | None | Food Game | 3.84 |
| Sodium Counter and Tracker for Healthy Food Diets | First Line Medical Communications Ltd | Paid | Nutrition Guide | 3.13 |
| Space Chef | The Lawrence Hall of Science | None | Food Game | 4.00 |
| SparkPeople Calorie Tracker | SparkPeople Inc. | None | Habit Trackers | 3.50 |
| Strawberry Ice Cream Sandwich | bweb media | None | Food Game | 3.46 |
| Strawberry Shortcake Bake Shop | Budge Studios | None | Food Game | 4.38 |
| Strawberry Shortcake Candy | Budge Studios | None | Food Game | 4.38 |
| Strawberry Shortcake Ice Cream* | Budge Studios | None | Food Game | 4.73 |
| Strawberry Shortcake Sweets* | Budge Studios | None | Food Game | 4.50 |
| Street Fair Food Cooking Kid Games (Girls & Boys) | App Whisperer LLC | None | Food Game | 3.35 |
| Street Food Kitchen Chef - Cooking Game | Girl Games - Vasco Games | None | Food Game | 3.54 |
| Strongr Fastr Workout, Meal and Diet Planner | Strongr Fastr LLC | None | Habit Trackers | 3.47 |
| Summer Food Cooking Maker Game | Best Fun Games | None | Food Game | 3.35 |
| Summer Slushy Maker â€“ Crazy Kids Food Making Games | Kids Crazy Games Media | None | Food Game | 3.54 |
| SuperFoodsRx - Essential Guide To Your Nutrition, Health & Wellness | SuperFoods Partners, LLC. | None | Nutrition Guide | 3.71 |
| Supermarket ‚Äì Game for Kids | Bubadu | None | Food Game | N/A |
| Supreme Pizza Maker - Kids Cooking Game* | Junior Games Studio | None | Food Game | 3.54 |
| Sushi Food Maker Cooking Kids Games (Girls & Boys)* | VZO entertainment | None | Food Game | 3.12 |
| Sweet Candy Maker Games* | Sunstorm Interactive | None | Food Game | 3.77 |
| Sweet Cookie Maker Kids Food | Appricot Studio - 2D Games | None | Food Game | 3.04 |
| Sweet Dessert Maker Games | Sunstorm Interactive | None | Food Game | 3.69 |
| Sweet Dippy Do! | Maverick Software LLC | Paid | Food Game | 3.23 |
| The Read and Feed app | EarthBound | None | Other | 3.43 |
| Toca Kitchen 2 | Toca Boca | None | Food Game | 4.46 |
| Toca Kitchen 2 | Toca Boca AB | Paid | Food Game | 3.93 |
| Toca Kitchen Monsters | Toca Boca AB | None | Food Game | 3.43 |
| Toddler Kitchen Food Cooking Games-EduKitchen Girl | Cubic FrogÂ® Apps-Learning Games for Kids | None | Food Game | 3.62 |
| Toddlers Food Games- EduKitchen | Cubic Frog Apps | None | Food Game | 3.50 |
| Tuberculosis TB Symptoms Causes & Diet Help | Kaveri Tyagi | None | Nutrition Guide | 3.16 |
| Unicorn Food - Cake Bakery | Kids Crazy Games Media | None | Food Game | 3.38 |
| Unicorn Food - Rainbow Glitter Food & Fashion* | TabTale | None | Food Game | 3.69 |
| Veeramachineni Rama Krishna Diet Program, VRK Diet | GLOBAL iMATRIX | None | Nutrition Guide | 3.38 |
| Vegan Diet | Gato Apps | None | Nutrition Guide | 3.43 |
| Vegetable Fun | BabyBus Kids Games | None | Food Game | N/A |
| Veggie Bottoms SD Healthy Eating Made Fun for Kids | Red Card Studios | None | Other | 3.53 |
| Vitamin B1,2,6 Counter & Tracker for Healthy Diets | First Line Medical Communications Ltd | Paid | Nutrition Guide | 3.13 |
| Vitamin C Counter & Tracker for Healthy Food Diets | First Line Medical Communications Ltd | Paid | Nutrition Guide | 3.09 |
| Vitamin E Counter & Tracker for Healthy Food Diets | First Line Medical Communications Ltd | Paid | Nutrition Guide | 3.13 |
| Vitamin K Counter & Tracker for Healthy Food Diets | First Line Medical Communications Ltd | Paid | Nutrition Guide | 3.09 |
| Vitamins A & D Counter & Tracker for Healthy Diets | First Line Medical Communications Ltd | Paid | Nutrition Guide | 3.13 |
| Vitamins B3, B5 Counter & Tracker for Healthy Diets | First Line Medical Communications Ltd | Paid | Nutrition Guide | 3.09 |
| Waffle Maker - Kids Cooking Food Salon Games* | Ninjafish Studios | None | Food Game | 3.46 |
| Water Drink Reminder | Leap Fitness Group | None | Habit Trackers | 3.46 |
| Weight Gain Diet Easy Safe Healthy Foods Plan Tips | SendGroupSMS.com Bulk SMS Software | None | Nutrition Guide | 3.00 |
| Weight Loss Diet Tips | Tototomato | None | Nutrition Guide | N/A |
| Whole Foods & Nutrition | ADKAApps | None | Nutrition Guide | 3.33 |
| Zinc Counter and Tracker for Healthy Food Diets | First Line Medical Communications Ltd | Paid | Nutrition Guide | 3.13 |
