# Supplementary material for: The Content, Quality, and Behavior Change Techniques in Nutrition-Themed Mobile Apps for Children in Canada: App Review and Evaluation Study
Source: JMIR Mhealth Uhealth. 2022 Feb 16;10(2):e31537. doi: 10.2196/31537 (PMC8892278; doi:10.2196/31537)
Supplement: Multimedia Appendix 3 [file mhealth_v10i2e31537_app3.docx]

**Multimedia Appendix 3.** Proportion of foods and beverages displayed in food game apps and non-game apps by food category (n=259)^a^

| **App Type** | **Game Apps (n = 162)** | **Non-Game Apps (n = 97)^b^** | ***P*-value*** |
| --- | --- | --- | --- |
| Fruit | 110 (67.9) | 90 (92.8) | *P*<.001 |
| Vegetables | 89 (54.9) | 90 (92.8) | *P*<.001 |
| Whole Grain Foods | 13 (8.0) | 68 (70.1) | *P*<.001 |
| Unprocessed Meats | 95 (58.6) | 80 (82.5) | *P*=.001 |
| Fish | 33 (20.4) | 71 (73.2) | *P*<.001 |
| Meat Alternatives | 29 (17.9) | 81 (83.5) | *P*<.001 |
| Milk Products | 110 (67.9) | 75 (77.3) | *P*=.14 |
| Milk Alternatives | 6 (3.7) | 32 (33.0) | *P*<.001 |
|  |  |  |  |
| Refined Grain Foods | 116 (71.6) | 35 (36.1) | *P*<.001 |
| Sugary Drinks | 62 (38.3) | 30 (30.9) | *P*=.29 |
| Desserts | 75 (46.3) | 28 (28.9) | *P*=.008 |
| Chocolate and Candies | 108 (66.7) | 25 (25.8) | *P*<0.001 |
| Salty Snacks | 38 (23.4) | 23 (23.7) | *P*=1 |
| Pizza | 29 (17.9) | 22 (22.7) | *P*=.44 |
| Fast Foods | 36 (22.2) | 21 (21.6) | *P*=1 |
| Processed Meats | 57 (35.2) | 24 (24.7) | *P*=.11 |

^a^Food categories were guided by the Canadian dietary guidelines (i.e., Canada’s Food Guide, Canada’s Dietary Guidelines). ^b^Non-game apps include nutrition guide, habit tracker and other. *Statistically significant at p<0.05 as determined by the χ^2^ test.
